# Time effect on cardiometabolic risk indicators in patients with bipolar disorder: a longitudinal case–control study

**DOI:** 10.1007/s00406-022-01520-7

**Published:** 2022-11-23

**Authors:** Hemen Najar, Erik Joas, Erik Pålsson, Mikael Landén

**Affiliations:** 1 Institute of Neuroscience and Physiology, Section of Psychiatry and Neurochemistry, Sahlgrenska Academy, University of Gothenburg, Blå Stråket 15, 413 45 Gothenburg, Sweden; 2 Department of Medical Epidemiology and Biostatistics, Karolinska Institutet, Stockholm, Sweden

**Keywords:** Bipolar disorder, Cardiovascular disease, Body mass index, Waist hip ratio, Blood pressure

## Abstract

Individuals with bipolar disorder are at increased risk for cardiovascular diseases. Most studies have described increases in cardiometabolic risk indicators (CMRIs) using clinical cut-off values. Further, there are no longitudinal studies on CMRIs. We aimed to investigate continuous measures of CMRIs in individuals with bipolar disorder and controls using both cross-sectional and longitudinal data. We used data from the Swedish St. Göran Bipolar project. Study individuals were examined at baseline and after a median of 6 and 7 years for the control and patient group, respectively. Data were collected December 2005–December 2020. The cohort included 281 individuals with bipolar disorder (mean age 39 years, 59% women) and 114 controls (mean age 38 years, 55% women). Of those, 155 patients and 74 controls also provided follow-up data. At baseline, individuals with bipolar disorder had significantly higher mean values of waist-to-hip ratio (WHR) (*β* = 0.142, *p* = 0.001), body mass index (*β* = 0.150, *p* = 0.006), plasma triacylglycerol (TAG) (*β* = 0.218, *p* < 0.001), total/plasma high-density lipoprotein-cholesterol (TChol/HDL-C) ratio (*β* = 0.103, *p* = 0.03), TAG/HDL-C ratio (*β* = 0.151, *p* = 0.006), and non-HDL-C (*β* = 0.168, *p* = 0.001) than controls. Most CMRIs remained higher in the patient group at follow-up. The difference between patients and controls increased over time for WHR (0.005 unit/year, *p* < 0.001), and systolic (1.1 mm Hg/year, *p* = 0.002) and diastolic (0.8 mm Hg/year, *p* < 0.001) blood pressure. Individuals with bipolar disorder displayed persistently higher levels of nearly all included CMRIs. Over time, a subset of CMRIs worsened in patients relative to controls. This suggests that active measures to counter cardiovascular risk in persons with bipolar disorder should be considered.

**Supplementary Information:**

The online version contains supplementary material available at 10.1007/s00406-022-01520-7.

## Introduction

In addition to the suffering from recurrent affective mood episodes, individuals with bipolar disorder are at increased risk for cardiovascular diseases (CVDs) [36]. Some CVD risk factors in bipolar disorder are shared with the general population [19]—albeit being more prevalent in individuals with bipolar disorder—such as smoking [27], sedentary lifestyle [27], alcohol use disorder [10], and unhealthy dietary habits [35]. Other CVD risk factors are specific for bipolar disorder, such as recurrent mood episodes [52], treatment with psychotropics—especially second-generation antipsychotic drugs [3]—as well as pleiotropic genes and biological pathways shared between bipolar disorder and cardiometabolic diseases [5]. Interactions between risk factors can increase CVD risk even further [29, 60].

General and bipolar-specific CVD risk factors mediate their effect on CVD partly through disturbances in lipid and glucose metabolism, overweight, and increased blood pressure [42, 46, 48, 58]. These cardiometabolic disturbances are gauged by various cardiometabolic risk indicators (CMRIs): Body mass index (BMI) along with waist-to-hip ratio (WHR) are indicators of total and central obesity, respectively, and associated with CVD risk [18]. Several lipoprotein ratios are used to optimize CVD risk prediction [45]. The ratio between total plasma cholesterol and plasma high-density lipoprotein-cholesterol (TChol/HDL-C ratio) and the ratio between plasma low-density lipoprotein-cholesterol (LDL-C) and HDL-C have greater predictive value than isolated biomarkers such as TChol or LDL-C [45].

Several studies have compared cardiometabolic disturbances in persons with bipolar disorder with the general population, but most have used clinical thresholds finding higher prevalence of total [43] and central obesity [22], dyslipidemia [44], diabetes [9], and hypertension [32] in bipolar disorder compared with the general population. Fewer studies have examined CMRIs as continuous variables. Some studies found higher BMI, waist circumference, systolic blood pressure (SBP), diastolic blood pressure (DBP), plasma triacylglycerol (TAG, formerly called triglyceride), and plasma glucose in bipolar disorder compared with the general population [8, 16, 24], but some found no difference regarding SBP [8, 24], DBP [8], TAG [8, 24], LDL-C [8], TChol [8], or fasting glucose in men [24], and conflicting data regarding HDL-C [8, 24]. There are no longitudinal studies on CMRIs in bipolar disorder other than for BMI.

Using clinical cut-off values of CMRIs instead of continuous data is a limitation, because CVD risk exists on a continuum and even subtle differences in CMRIs are associated with higher CVD risk [13, 14, 20, 47, 59]. Moreover, the cut-offs could reflect extreme values [40]. And expert committees are not in agreement and have repeatedly revised the specified thresholds for defining hypertension [26], diabetes [21], dyslipidemia [6, 57], and obesity [4].

The aim of this study was to investigate continuous measures of CMRIs in individuals with bipolar disorder and controls using both cross-sectional and longitudinal data.

## Methods

### Study population and ethical approval

We used data from the St. Göran Bipolar project that started in 2005. This is a naturalistic longitudinal clinical study of individuals with bipolar syndromes and controls. Study patients were enrolled at a bipolar tertiary outpatient clinic in Stockholm, Sweden. A semi-structured diagnostic interview was performed using a Swedish version of the Affective Disorder Evaluation (ADE), which was originally developed for the Systematic Treatment Enhancement Program of Bipolar Disorder (STEP-BD) [53]. The ADE includes the affective module in SCID-I (Structured Clinical Interview for DSM-IV Axis I Disorders), as well as a social anamnesis, information on the number lifetime affective episodes, tobacco use, alcohol and drug use, childhood and family history, information on violent behavior, suicide attempts, somatic illness, and reproductive history. Scores from both the symptom and function domains of the Global Assessment of Functioning (GAF) scales were used to assess the overall psychosocial functioning [33]. The Mini International Neuropsychiatric Interview (M.I.N.I.) was used to screen for psychiatric diagnoses other than bipolar disorder [56]. A final best estimate diagnosis was set at a diagnostic case conference by psychiatrists specialized in bipolar disorder.

The inclusion criteria for the St. Göran Bipolar study were age ≥ 18 years and meeting the DSM-IV criteria for any bipolar spectrum disorder, i.e., type I, II, non-otherwise specified (NOS), cyclothymia, or schizoaffective syndrome bipolar type. Patients were excluded if they were unable to complete the standard clinical assessment or were incapable of providing informed consent. Psychiatric and somatic comorbidities were not exclusion criteria. In this study, individuals with schizoaffective syndrome bipolar type were excluded from analysis.

For every patient that had been included when the recruitment of control individuals started, Statistics Sweden (SCB) randomly selected seven age- and sex-matched population-based controls living in the same catchment area as the patients and contacted them by mail. Those who responded were first interviewed over phone by research nurses who screened for the exclusion criteria: any current psychiatric disorder or any current use of psychotropic drugs, bipolar disorder or schizophrenia in first degree relatives, neurological diseases (excluding mild migraine), untreated endocrine disorders, pregnancy, substance or alcohol abuse. Controls were then scheduled for an appointment where an M.I.N.I interview was completed by a psychiatrist to exclude psychiatric disorders. The Alcohol Use Disorders Identification Test (AUDIT) [55] and the Drug Use Disorders Identification Test (DUDIT) [7] were used to screen for alcohol and drug use disorder, respectively.

Study participants were recontacted after a median of 7 years (ranging from 4–11 years) for the patient group and a median of 6 years (ranging from 5–7 years) for the control group. The follow-up visit followed the same procedures regarding physical examination and blood testing as the baseline visit. Baseline examinations were conducted December 2005–June 2015. Follow-up examinations were conducted April 2012–December 2020.

The study was approved by the regional ethical review board in Stockholm, Sweden (registration code: 2005/554–31/3). All participants consented orally and in writing after being presented with detailed information about the study.

### Physical measurements and laboratory analyses

Blood was sampled in the morning after an overnight fast. All plasma lipid analyses were performed at the Unilabs laboratory, St. Göran Hospital, Stockholm by enzymatic photometry (Siemens Advia XPT). Waist circumference was measured midway between the lower rib and the anterior superior iliac spine at the umbilical level in a fasting state. Hip circumference was measured around the widest portion of the gluteal region and hip. Left arm blood pressure was measured in supine position, after resting for 15 min, using a manual sphygmomanometer (H-E AB cuff size 12 × 35). A larger/appropriately sized cuff was available when needed. Weight was measured with the participant having light clothes and no shoes. Height was self-reported. BMI was calculated as the weight in kilograms divided by the square of the height in meters (kg/m^2^). Weight, height, waist circumference, hip circumference, and blood pressure were measured to the nearest whole kg, cm, or mm Hg according to clinical praxis. Despite this, some measurements were entered with one decimal point: waist circumference (*n* = 5 at baseline), hip circumference (*n* = 3 at baseline), and weight (*n* = 2 at follow-up), and height (*n* = 4 at follow-up). For consistency, we rounded those 14 measurements to the nearest whole number prior to analysis. All current use of medications was recorded.

We excluded fasting plasma glucose and LDL-C from case–control comparisons, because measurement routines at the laboratory changed during the study. As a substitute for fasting glucose, we used the ratio between fasting TAG and HDL-C (TAG/HDL-C ratio). This ratio is an established surrogate measure of plasma atherogenicity [45] and insulin resistance [23]. Lacking LDL-C, we were unable to calculate LDL-C/HDL-C ratio. Instead, we calculated the TChol/HDL-C ratio, which in fact gives a better prediction of CVD risk than the LDL-C/HDL-C ratio [41]. Furthermore, we used non-HDL-C level, which is a better predictor of cardiovascular events than LDL-C level [17]. Non-HDL-C is an aggregate measure that includes the concentrations of all atherogenic cholesterol present in apolipoprotein B-containing lipoproteins: very low-density lipoprotein, intermediate-density lipoprotein, chylomicron remnants, lipoprotein (a), and LDL-C. We calculated non-HDL-C by subtracting HDL-C from TChol.

### Statistical analysis

All analyses were conducted using SPSS Statistics (version 28). We used independent sample *t* tests and linear regression models to examine group differences.

To decrease the risk for false positives due to multiple testing, we restricted comparisons to the following CMRIs of high clinical relevance: WHR, BMI, SBP, DBP, TAG, TAG/HDL-C ratio, TChol/HDL-C ratio, and non-HDL-C. Moreover, we applied *p* value correction according to Hochberg for the above eight tests [30].

We found that all CMRIs had some missing values and, therefore, performed a missing data analysis. At baseline, 10.7% in the patient group and 3.5% in the control group had some missing data on CMRIs. At follow-up, the numbers were 11.6% and 4.1% in the patient and control group, respectively. We plotted the date of observation against the missing values of the CMRIs (results not shown). We found that more data were missing in the beginning of both observation periods (baseline and follow-up) indicating that the probability of missingness at least partly depended on the date of observation [50]. We could not identify any plausible missing not at random (MNAR) mechanism and assumed a missing at random (MAR) mechanism of missingness [50].

Given more than 10% missing data in some groups, and that data presumably were missing at random, we opted for multiple imputation in dealing with missing data [50]. Multiple imputation provides more accurate standard errors for hypothesis testing and less biased parameter estimates like means, standard deviations, regression coefficients, and correlations. We performed two sets of imputations: one for baseline data and one for follow-up data. We adopted an inclusive strategy [12] where a list of observed/auxiliary variables were included in the imputation model to improve the performance of multiple imputation, decrease the probability of omitting important causes of missing data, increase efficiency of statistical inferences related to CMRIs, and increase the statistical power [12, 50]. These auxiliary variables were sex, age, having bipolar disorder, number of cigarettes smoked per day, more than 12 years of education, working at least 50%, GAF-scores, weight, height, hip circumference, waist circumference, SBP, DBP, TChol, TAG, HDL-C, somatic illness, treatment of diabetes and hypothyroidism, and treatment with psychotropics (including lithium, valproate, lamotrigine, antidepressants, first- and second-generation antipsychotics, and central stimulants), lipid lowering agents, and antihypertensives. Five imputed datasets were generated using predictive mean-matching methods.

We used linear mixed effects model with a random intercept to examine the difference in trajectories between patients and controls across the follow-up for the different CMRIs. We adjusted the linear mixed effects model for sex, age at baseline, and for follow-up time. We adjusted for follow-up time as this varied both within and between groups. We considered a two tailed and Hochberg-corrected *p* < 0.05 as statistically significant.

## Results

The dataset included 325 patients and 115 controls at baseline. We excluded 2 individuals with schizoaffective syndrome bipolar type and 42 patients due to total missing of CMRIs´ data and hence, we were unable to use the data from these individuals in the multiple imputation model [50]. We excluded one control due to total missing of CMRIs´ data. The final baseline cohort, hence, included 281 patients and 114 controls. The longitudinal dataset comprised 155 patients and 74 controls who participated at both baseline and follow-up.

The clinical characteristics of the study groups at baseline are outlined in Table 1. Individuals with bipolar disorder had lower GAF-scores, lower prevalence of working at least 50%, lower prevalence of education > 12 years, higher prevalence of smoking across three categories (light, moderate, and heavy smokers), and higher prevalence of somatic comorbidities compared with the controls.

**Table 1:** Clinical characteristics of the study groups at baseline

	Patients	*n*	Controls	*n*
Women, *n* (%)	165 (59)	281	63 (55)	114
Age, mean ± SD, years	39 ± 13	281	38 ± 13	114
GAF symptom, mean ± SD	67 ± 10	258	79 ± 6	110
GAF function, mean ± SD	67 ± 11	258	79 ± 6	112
Smoking		259		114
Non-smoker, *n* (%)	177 (68.3)		99 (86.8)	
Light smoker (< 10 cigarettes per day), *n* (%)	30 (11.6)		11 (9.6)	
Moderate smoker (10–19 cigarettes per day), *n* (%)	32 (12.4)		3 (2.6)	
Heavy smoker (≥ 20 cigarettes per day), *n* (%)	20 (7.7)		1 (0.9)	
> 12 y of education, *n* (%)	147 (57)	260	71 (62)	114
Working more than 50%, *n* (%)	160 (63)	253	100 (88)	114
Somatic comorbidity				
Hypertension, *n* (%)	15 (5.7)	261	3 (2.6)	114
Angina pectoris, *n* (%)	5 (1.9)	261	0	114
Myocardial infarction, *n* (%)	4 (1.5)	261	0	114
Other heart problems, *n* (%)	2 (0.8)	261	0	114
Cerebrovascular disease, *n* (%)	1 (0.4)	261	1 (0.9)	114
Migraine, *n* (%)	28 (10.7)	261	4 (3.5)	114
Diabetes mellitus type II, *n* (%)	5 (1.9)	262	1 (0.9)	114
Diabetes mellitus type I, *n* (%)	1 (0.4)	261	0	114
Hypothyroidism, *n* (%)	38 (14.4)	264	1 (0.9)	114
Hyperthyroidism, *n* (%)	5 (1.9)	261	0	114
Disease duration, median (25–75 percentiles), years	16 (9–23)	259		
Age at first treatment with psychotropics, mean ± SD, years	29 ± 11	173		
Bipolar subtype, *n* (%)		281		
Bipolar I disorder	160 (57)			
Bipolar II disorder	96 (34)			
Bipolar disorder non-otherwise specified (NOS)	23 (8)			
Cyclothymia	2 (1)			
Prescribed psychotropics, *n* (%)		281		
Lithium	159 (57)			
Valproate	58 (21)			
Lamotrigine	35 (13)			
Antidepressants	113 (40)			
Antipsychotics (FGA and SGA)	72 (26)			
Central stimulants	19 (7)			

*FGA* first-generation antipsychotics, *GAF* global assessment of functioning, *SD* standard deviation, *SGA* second-generation antipsychotics

### Baseline

Table 2 shows the case–control comparisons at baseline. After adjusting for age and sex, and correcting for multiple comparisons, individuals with bipolar disorder had significantly higher mean WHR (*β* = 0.142, *p* = 0.001), BMI (*β* = 0.150, *p* = 0.006), TAG (*β* = 0.218, *p* < 0.001), TAG/HDL-C ratio (*β* = 0.151, *p* = 0.006), TChol/HDL-C ratio (*β* = 0.103, *p* = 0.03), and non-HDL-C (*β* = 0.168, *p* = 0.001) than controls. Regarding blood pressure, however, the mean SBP (*β* =  −0.233, *p* < 0.001) and DBP (*β* =  −0.108, *p* = 0.03) were higher in the control group.

**Table 2:** Baseline comparisons of cardiometabolic risk indicators between patients and controls

CMRIs	Patients (*n* = 281)	Controls (*n* = 114)	*t* test		Linear regression (adjusted for age and sex)	
			Mean difference (95% CI)	*p* value*	Coefficient estimate	*p* value*
WHR, mean ± SD	0.86 ± 0.09	0.83 ± 0.09	0.03 (0.008–0.05)	0.02	0.14	0.001
BMI, mean ± SD, kg/m^2^	25.5 ± 4.5	24.1 ± 3.8	1.5 (0.6–2.3)	0.004	0.15	0.006
SBP, mean ± SD, mm Hg	117.7 ± 14.9	125.7 ± 15.8	−7.9 (−11.3 to −4.7)	< 0.001	−0.23	< 0.001
DBP, mean ± SD, mm Hg	77.4 ± 9.3	79.5 ± 8.1	−2.1 (−3.9 to −0.2)	0.047	−0.11	0.03
TAG, mean ± SD, mmol/L	1.2 ± 0.9	0.9 ± 0.5	0.4 (0.2–0.5)	< 0.001	0.22	< 0.001
TAG/HDL-C ratio, mean ± SD	0.9 ± 0.9	0.7 ± 0.6	0.3 (0.1–0.4)	0.004	0.15	0.006
TChol/HDL-C ratio, mean ± SD	3.6 ± 1.1	3.3 ± 1.1	0.2 (0.003–0.5)	0.047	0.10	0.03
Non-HDL-C, mean ± SD, mmol/L	3.6 ± 1.1	3.2 ± 0.9	0.4 (0.2–0.7)	0.003	0.17	0.001

Comparisons are made using multiply imputed data

*BMI* body mass index, *CI* confidence interval, *CMRIs* cardiometabolic risk indicators, *DBP* diastolic blood pressure, *HDL-C* plasma high-density lipoprotein-cholesterol, *SBP* systolic blood pressure, *SD* standard deviation, *TAG* fasting plasma triacylglycerol, *TChol* total plasma cholesterol, *WHR* waist-to-hip ratio

*Corrected for multiple comparisons

To examine the performance of the multiple imputation, we conducted a sensitivity analysis with available cases. Supplementary Table 1 shows that the estimates of mean values and standard deviations were preserved in this sensitivity analysis.

Further, we performed a sensitivity analysis excluding individuals who were on treatment that could directly affect CMRIs, i.e., pharmacological treatment for dyslipidemia (9 patients and 2 controls) or hypertension (12 patients and 3 controls). The results shown in Supplementary Table 2 were similar to the results in Table 2.

We also compared the mean levels of CMRIs in patients who participated at baseline only with patients who participated at both baseline and follow-up. Results were adjusted for age and sex, and *p* values were corrected for multiple comparisons. We found no differences in the mean levels of CMRIs between these patient-groups (Supplementary Table 3). On the other side, we found no differences between controls that participated at baseline only and controls who participated at both baseline and follow-up (Supplementary Table 4).

Finally, we compared baseline data between patients and controls for those who participated at baseline and follow-up (Supplementary Table 5). We replicated all statistically significant results from the analysis of the whole baseline dataset except for TChol/HDL-C ratio and DBP.

### Follow-up

At follow-up, we found statistically significant higher mean levels of WHR (*β* = 0.290, *p* < 0.001), BMI (*β* = 0.161, *p* = 0.045), TAG (*β* = 0.190, *p* = 0.02), TAG/HDL-C ratio (*β* = 0.193, *p* = 0.02), TChol/HDL-C ratio (*β* = 0.199, *p* = 0.007), and DBP (*β* = 0.157, *p* = 0.04) in the patient group than the control group, whereas the mean level of SBP (*β* = 0.015, *p* > 0.30) and non-HDL-C (*β* = 0.138, *p* = 0.05) did not differ significantly between the two groups (Supplementary Table 6).

### Time-group interaction

**Fig. 1:**
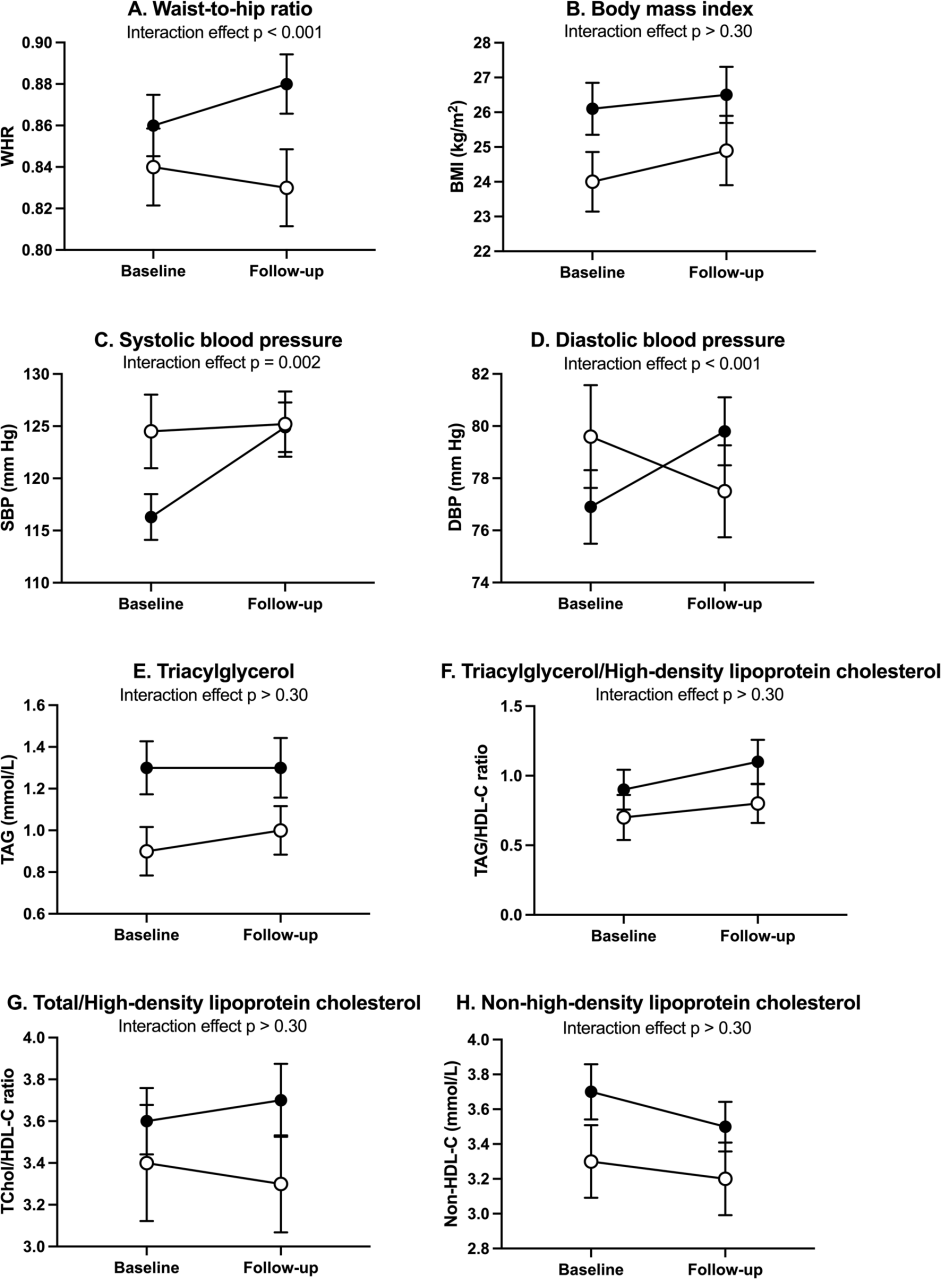
CMRI levels at baseline and follow-up in individuals with bipolar disorder (filled circles) and controls (open circles). Data are presented as means and 95% CI, comparisons are made using multiply imputed data. The significance values for the interaction effect in a linear mixed effects model were adjusted for age at baseline, sex, and follow-up time and corrected for multiple comparisons. *BMI* body mass index, *DBP* diastolic blood pressure, *SBP* systolic blood pressure, *TAG* fasting plasma triacylglycerol, *HDL-C* plasma high-density lipoprotein-cholesterol, *TChol* total plasma cholesterol, *WHR* waist-to-hip ratio

We included only those who participated at both baseline and follow-up in the linear mixed effects model. We tested time-group interaction effect between patients and controls while adjusting for follow-up time and with and without adjusting for sex and baseline value of age. *p* values were corrected for multiple comparisons (Fig. 1A–H and Supplementary Table 7). The difference in average annual change between the patient and the control group indicated an increase in patients relative to controls over time in WHR (0.005 unit/year, *p* < 0.001), SBP (1.1 mm Hg/year, *p* = 0.002), and DBP (0.8 mm Hg/year, *p* < 0.001). The time-group interaction was not statistically significant for BMI, TAG, TAG/HDL-C ratio, TChol/HDL-C ratio, and non-HDL-C. We examined the performance of multiple imputation in the latter analyses by executing an available case sensitivity analysis (Supplementary Table 8). The coefficient estimates were preserved in this sensitivity analysis.

## Discussion

In this first longitudinal study on CMRIs including lipoprotein ratios in bipolar disorder, we first found higher mean baseline values for measures of total and central obesity, and atherogenic lipid profile in individuals with bipolar disorder compared with a control group. Second, we found that most CMRIs´ differences between patients and controls remained after a median follow-up of 6–7 years. Third, we found interaction effects for central obesity and blood pressure indicating a worsening in the bipolar disorder group relative to controls during the follow-up period.

The observed group-level differences in the CMRIs and the changes over time are of clinical significance despite small effect sizes. For example, we found 0.03 units higher WHR and 1.5 kg/m^2^ higher BMI in patients compared with controls at baseline. Previous studies have shown an increased risk of CVD by 5 percent for every 0.01 increase in WHR [20] and for every 1 kg/m^2^ increase in BMI [47]. Likewise, we found that patients had 0.4 mmol/L higher mean non-HDL-C and 0.2 units higher TChol/HDL-C ratio than controls at baseline. Previous studies have found that a 0.78 mmol/L increase in non-HDL-C, or a 1-unit increase in TChol/HDL-C ratio, are associated with increased risk for CVD death by 15–19 percent [14, 17]. Conversely, we found 0.4 mmol/L higher mean TAG in patients than controls at baseline, and lowering TAG by only 0.1 mmol/L can reduce coronary events by 5 percent [34]. Finally, the patient group had 0.3 units higher TAG/HDL-C ratio compared with controls at baseline. Small increases in TAG/HDL-C ratio—from 0.1 unit—can in addition to increasing the risk for CVD, increase the risk for insulin resistance [25, 28].

Systolic and diastolic blood pressure were somewhat surprisingly lower in patients than controls at baseline. This finding is at odds with most previous studies that have found higher prevalence of hypertension in individuals with bipolar disorder compared with controls [19]. However, there are also studies that have reported no difference [11] or higher blood pressure in healthy controls than individuals with bipolar disorder [49]. We saw no evidence of systematic variation or bias regarding the measurement of blood pressure (results not shown). One potential explanation is the effect of white coat syndrome in the control group [38, 51], which consists of healthy individuals with less previous contact with health care personnel than patients. Over the study period, SBP and DBP increased in the patient group by 8.6 mm Hg and 2.9 mm Hg, respectively. Previous studies have reported an increase in CVD risk of up to 53 percent with every 10-mm Hg increase in SBP [59]. Conversely, lowering DBP by 2 mm Hg can result in a 6 percent reduction in the risk of coronary heart disease and a 15 percent reduction in risk of stroke and transient ischemic attacks [13].

WHR increased over time in patients relative to controls which confirms the tendency of individuals with bipolar disorder to a more central type of obesity [37]. However, the time-group interaction was not statistically significant for total obesity as measured by BMI. We cannot exclude a possible selection bias because the patients who participated at baseline and follow-up had numerically higher—although not statistically significant—BMI than those patients who only participated at baseline. Furthermore, we lack information to determine the relative contribution of muscle and fat mass to increases in BMI. It is, however, worth noticing that CVD risk associated with central obesity (i.e., WHR) is independent of BMI [54].

A striking finding in this study was that individuals with bipolar disorder differed from controls across the whole range of CMRIs. This is concerning, because studies have shown synergistic effects from multiple CMRIs, producing a higher combined risk than simply summarizing each risk indicator [31, 40]. Given that the patients had a mean age of 39 years at baseline, it is also worrying that some CMRIs continued to increase in patients during the study. Persons with bipolar disorder have a shorter life span by 8.5 to 12.7 years compared with the general population in Sweden [15, 39]. The higher CMRIs levels in persons with bipolar disorder, and the continuous increase in a subset of CMRIs relative to controls, are likely contributing factors.

### Strengths and limitations

The strengths of our study include the longitudinal study design where we followed a large cohort of individuals with bipolar disorder and controls during 6–7 years. The phenotyping was meticulous and included relevant auxiliary variables that reduce bias. We applied the less biased/less error-prone technique of multiple imputation in dealing with missing values [50].

This study also has some limitations. We cannot determine the cause of higher CMRIs in patients or the association of individual CVD risk factors with CMRIs due to the study design. There is a potential selection bias in our study cohort. Both patients and controls had a lower prevalence of obesity (BMI ≥ 30 kg/m^2^) at baseline compared with the respective population estimates. The prevalence of obesity was 6 percent in the control group at baseline (results not shown), which should be compared with an estimate of 16 percent in the Swedish general population 2020 [2]. Likewise, the prevalence of obesity was 14.5 percent in our bipolar disorder group (results not shown), which can be compared with 32.6 percent in the Swedish National Quality Register for Bipolar Disorder 2020 [1]. A potential explanation is that our cohort was recruited in the Stockholm metropolitan area, limiting the generalizability to the rest of Sweden. Selection bias is also a potential problem in the longitudinal analysis since not all participants were available for follow-up. Finally, although we found no evidence of bias in the baseline blood pressure estimates, the finding that patients had lower blood pressure at baseline conflicts with previous studies. The increase during follow-up in the patient group might, therefore, be explained by regression to the mean and should be interpreted with caution.

## Conclusion

Individuals with bipolar disorder had persistently higher mean values for nearly all included CMRIs compared with a control group. Further, central obesity and blood pressure worsened in patients relative to controls over the follow-up period. Despite that the case–control differences were small, several previous studies have shown that minor increases in CMRIs, and the synergism between CMRIs, have clinically significant effects on CVD risk and mortality. In summary, the study shows that persons with bipolar disorder are at increased risk for CVD. Cardiovascular risk management programs including early intervention with weight decreasing programs and lipid and blood pressure lowering medications should preferably be integrated in the psychiatric health care system to reduce CVD risk in persons with bipolar disorder.

## Supplementary Information

Below is the link to the electronic supplementary material.

Supplementary file1 (DOCX 23 KB)

Supplementary file2 (DOCX 22 KB)

Supplementary file3 (DOCX 22 KB)

Supplementary file4 (DOCX 22 KB)

Supplementary file5 (DOCX 22 KB)

Supplementary file6 (DOCX 24 KB)

Supplementary file7 (DOCX 23 KB)

Supplementary file8 (DOCX 23 KB)

## Data Availability

The data that support the findings of this study are archived at University of Gothenburg and availability is regulated by Swedish law.
