# Supplementary material for: Time effect on cardiometabolic risk indicators in patients with bipolar disorder: a longitudinal case–control study
Source: Eur Arch Psychiatry Clin Neurosci. 2022 Nov 23;273(5):1191–200. doi: 10.1007/s00406-022-01520-7 (PMC10359211; doi:10.1007/s00406-022-01520-7)
Supplement: Supplementary file 2 — Supplementary file2 (DOCX 22 KB) [file 406_2022_1520_MOESM2_ESM.docx]

**Supplementary table 2. A sensitivity analysis excluding those individuals taking treatment that could directly affect the specific CMRI at baseline**

| CMRIs | Patients (n=263) | Controls (n=109) | T-test | | Linear regression  (adjusted for age and sex) | |
| --- | --- | --- | --- | --- | --- | --- |
|  |  |  | **Mean difference (95% CI)** | **P-value**^^ | **Coefficient estimate** | **P-value**^^ |
| WHR, mean ± SD | 0.85 ± 0.09 | 0.83 ± 0.09 | 0.03 (0.006 – 0.05) | 0.03 | 0.14 | 0.002 |
| BMI, mean ± SD, kg/m^2^ | 25.3 ± 4.3 | 24.1 ± 3.8 | 1.2 (0.3 – 2.1) | 0.04 | 0.13 | 0.03 |
| SBP, mean ± SD, mm Hg | 116.9 ± 14.6 | 124.3 ± 14.2 | - 7.4 (- 10.6 – - 4.2) | < 0.001 | - 0.22 | < 0.001 |
| DBP, mean ± SD, mm Hg | 76.8 ± 9.1 | 78.9 ± 7.6 | - 2.1 (- 3.9 – - 0.2) | 0.05 | - 0.11 | 0.03 |
| TAG, mean ± SD, mmol/L | 1.2 ± 0.8 | 0.9 ± 0.5 | 0.3 (0.2 – 0.5) | < 0.001 | 0.22 | < 0.001 |
| TAG/HDL-C ratio, mean ± SD | 0.9 ± 0.8 | 0.7 ± 0.6 | 0.2 (0.05 – 0.4) | 0.04 | 0.14 | 0.02 |
| TChol/HDL-C ratio, mean ± SD | 3.6 ± 1.1 | 3.3 ± 1.1 | 0.2 (- 0.001 – 0.5) | 0.05 | 0.11 | 0.03 |
| Non-HDL-C, mean ± SD, mmol/L | 3.6 ± 1.1 | 3.2 ± 0.9 | 0.4 (0.2 – 0.7) | 0.001 | 0.18 | < 0.001 |
| * Corrected for multiple comparisons.  Note  Comparisons are made using multiply imputed data.  Abbreviations: BMI, body mass index; CI, confidence interval; CMRIs, cardiometabolic risk indicators; DBP, diastolic blood pressure; HDL-C, plasma high-density lipoprotein-cholesterol; SBP, systolic blood pressure; SD, standard deviation; TAG, fasting plasma triacylglycerol; TChol, total plasma cholesterol; WHR, waist-to-hip ratio. | | | | | | |
