# Supplementary material for: Time effect on cardiometabolic risk indicators in patients with bipolar disorder: a longitudinal case–control study
Source: Eur Arch Psychiatry Clin Neurosci. 2022 Nov 23;273(5):1191–200. doi: 10.1007/s00406-022-01520-7 (PMC10359211; doi:10.1007/s00406-022-01520-7)
Supplement: Supplementary file 3 — Supplementary file3 (DOCX 22 KB) [file 406_2022_1520_MOESM3_ESM.docx]

**Supplementary table 3. Baseline comparison of cardiometabolic risk indicators between patients who participated in follow-up with patients who didn´t**

| CMRIs | Participated at baseline and follow-up (n=155) | Participated at baseline only (n=126) | T-test | | Linear regression  (adjusted for age and sex) | | |
| --- | --- | --- | --- | --- | --- | --- | --- |
|  |  |  | **Mean difference (95% CI)** | **P-value**^*^ | **Coefficient estimate** | | **P-value**^*^ |
| WHR, mean ± SD | 0.86 ± 0.09 | 0.86 ± 0.09 | - 0.01 (- 0.03 – 0.02) | > 0.30 | 0.01 | > 0.30 | |
| BMI, mean ± SD, kg/m^2^ | 26.1 ± 4.7 | 24.8 ± 4.2 | 1.3 (0.2 – 2.4) | 0.1 | 0.15 | 0.07 | |
| SBP, mean ± SD, mm Hg | 116.3 ± 13.8 | 119.5 ± 16.1 | - 3.2 (- 6.7 – 0.3) | > 0.30 | - 0.07 | > 0.30 | |
| DBP, mean ± SD, mm Hg | 76.9 ± 8.9 | 77.9 ± 9.7 | - 1.1 (- 3.3 – 1.1) | > 0.30 | - 0.03 | > 0.30 | |
| TAG, mean ± SD, mmol/L | 1.3 ± 0.8 | 1.2 ± 0.9 | 0.04 (- 0.2 – 0.2) | > 0.30 | 0.04 | > 0.30 | |
| TAG/HDL-C ratio, mean ± SD | 0.9 ± 0.9 | 0.9 ± 0.9 | 0.07 (- 0.1 – 0.3) | > 0.30 | 0.06 | > 0.30 | |
| TChol/HDL-C ratio, mean ± SD | 3.6 ± 1.0 | 3.5 ± 1.1 | 0.1 (- 0.1 – 0.4) | > 0.30 | 0.08 | > 0.30 | |
| Non-HDL-C, mean ± SD, mmol/L | 3.7 ± 1.0 | 3.6 ± 1.2 | 0.1 (- 0.2 – 0.4) | > 0.30 | 0.06 | > 0.30 | |
| * Corrected for multiple comparisons.  Note  Comparisons are made using multiply imputed data.  Abbreviations: BMI, body mass index; CI, confidence interval; CMRIs, cardiometabolic risk indicators; DBP, diastolic blood pressure; HDL-C, plasma high-density lipoprotein-cholesterol; SBP, systolic blood pressure; SD, standard deviation; TAG, fasting plasma triacylglycerol; TChol, total plasma cholesterol; WHR, waist-to-hip ratio. | | | | | | | |
