# Supplementary material for: Time effect on cardiometabolic risk indicators in patients with bipolar disorder: a longitudinal case–control study
Source: Eur Arch Psychiatry Clin Neurosci. 2022 Nov 23;273(5):1191–200. doi: 10.1007/s00406-022-01520-7 (PMC10359211; doi:10.1007/s00406-022-01520-7)
Supplement: Supplementary file 4 — Supplementary file4 (DOCX 22 KB) [file 406_2022_1520_MOESM4_ESM.docx]

**Supplementary table 4. Baseline comparison of cardiometabolic risk indicators between controls who participated in follow-up with controls who didn´t**

| CMRIs | Participated at baseline and follow-up (n=74) | Participated at baseline only (n=40) | T-test | | Linear regression  (adjusted for age and sex) | |
| --- | --- | --- | --- | --- | --- | --- |
|  |  |  | **Mean difference (95% CI)** | **P-value**^*^ | **Coefficient estimate** | **P-value**^*^ |
| WHR, mean ± SD | 0.84 ± 0.08 | 0.82 ± 0.09 | 0.02 (- 0.01 – 0.06) | > 0.30 | 0.02 | > 0.30 |
| BMI, mean ± SD, kg/m^2^ | 24.0 ± 3.7 | 24.2 ± 3.9 | - 0.2 (- 1.7 – 1.3) | > 0.30 | - 0.05 | > 0.30 |
| SBP, mean ± SD, mm Hg | 124.5 ± 15.2 | 127.9 ± 16.9 | - 3.4 (- 9.5 – 2.7) | > 0.30 | - 0.19 | 0.2 |
| DBP, mean ± SD, mm Hg | 79.6 ± 8.5 | 79.2 ± 7.4 | 0.4 (- 2.7 – 3.6) | > 0.30 | - 0.07 | > 0.30 |
| TAG, mean ± SD, mmol/L | 0.9 ± 0.5 | 0.8 ± 0.4 | 0.1 (- 0.1 – 0.3) | > 0.30 | 0.09 | > 0.30 |
| TAG/HDL-C ratio, mean ± SD | 0.7 ± 0.7 | 0.6 ± 0.4 | 0.1 (- 0.1 – 0.4) | > 0.30 | 0.09 | > 0.30 |
| TChol/HDL-C ratio, mean ± SD | 3.4 ± 1.2 | 3.1 ± 0.9 | 0.3 (- 0.2 – 0.7) | > 0.30 | 0.07 | > 0.30 |
| Non-HDL-C, mean ± SD, mmol/L | 3.3 ± 0.9 | 3.0 ± 0.8 | 0.3 (- 0.1 – 0.7) | > 0.30 | 0.05 | > 0.30 |
| * Corrected for multiple comparisons.  Note  Comparisons are made using multiply imputed data.  Abbreviations: BMI, body mass index; CI, confidence interval; CMRIs, cardiometabolic risk indicators; DBP, diastolic blood pressure; HDL-C, plasma high-density lipoprotein-cholesterol; SBP, systolic blood pressure; SD, standard deviation; TAG, fasting plasma triacylglycerol; TChol, total plasma cholesterol; WHR, waist-to-hip ratio. | | | | | | |
