# Supplementary material for: Time effect on cardiometabolic risk indicators in patients with bipolar disorder: a longitudinal case–control study
Source: Eur Arch Psychiatry Clin Neurosci. 2022 Nov 23;273(5):1191–200. doi: 10.1007/s00406-022-01520-7 (PMC10359211; doi:10.1007/s00406-022-01520-7)
Supplement: Supplementary file 5 — Supplementary file5 (DOCX 22 KB) [file 406_2022_1520_MOESM5_ESM.docx]

**Supplementary table 5. Baseline comparison of cardiometabolic risk indicators between patients and controls who participated at baseline and follow-up**

| CMRIs | Patients (n=155) | Controls (n=74) | T-test | | Linear regression  (adjusted for age and sex) | |
| --- | --- | --- | --- | --- | --- | --- |
|  |  |  | **Mean difference (95% CI)** | **P-value**^*^ | **Coefficient estimate** | **P-value**^*^ |
| WHR, mean ± SD | 0.86 ± 0.09 | 0.84 ± 0.08 | 0.02 (- 0.008 – 0.04) | 0.2 | 0.15 | 0.008 |
| BMI, mean ± SD, kg/m^2^ | 26.1 ± 4.7 | 24.0 ± 3.7 | 2.1 (0.9 – 3.2) | 0.002 | 0.23 | 0.002 |
| SBP, mean ± SD, mm Hg | 116.3 ± 13.8 | 124.5 ± 15.2 | - 8.2 (- 12.2 – - 4.3) | < 0.001 | - 0.22 | < 0.001 |
| DBP, mean ± SD, mm Hg | 76.9 ± 8.9 | 79.6 ± 8.5 | - 2.7 (- 5.2 – - 0.3) | 0.1 | - 0.10 | 0.08 |
| TAG, mean ± SD, mmol/L | 1.3 ± 0.8 | 0.9 ± 0.5 | 0.4 (0.2 – 0.5) | 0.002 | 0.24 | 0.001 |
| TAG/HDL-C ratio, mean ± SD | 0.9 ± 0.9 | 0.7 ± 0.7 | 0.2 (0.009 – 0.5) | 0.1 | 0.16 | 0.04 |
| TChol/HDL-C ratio, mean ± SD | 3.6 ± 1.0 | 3.4 ± 1.2 | 0.2 (- 0.1 – 0.5) | 0.2 | 0.12 | 0.08 |
| Non-HDL-C, mean ± SD, mmol/L | 3.7 ± 1.0 | 3.3 ± 0.9 | 0.4 (0.1 – 0.6) | 0.06 | 0.19 | 0.005 |
| * Corrected for multiple comparisons.  Note  Comparisons are made using multiply imputed data.  Abbreviations: BMI, body mass index; CI, confidence interval; CMRIs, cardiometabolic risk indicators; DBP, diastolic blood pressure; HDL-C, plasma high-density lipoprotein-cholesterol; SBP, systolic blood pressure; SD, standard deviation; TAG, fasting plasma triacylglycerol; TChol, total plasma cholesterol; WHR, waist-to-hip ratio. | | | | | | |
