# Supplementary material for: Time effect on cardiometabolic risk indicators in patients with bipolar disorder: a longitudinal case–control study
Source: Eur Arch Psychiatry Clin Neurosci. 2022 Nov 23;273(5):1191–200. doi: 10.1007/s00406-022-01520-7 (PMC10359211; doi:10.1007/s00406-022-01520-7)
Supplement: Supplementary file 6 — Supplementary file6 (DOCX 24 KB) [file 406_2022_1520_MOESM6_ESM.docx]

**Supplementary table 6. Follow-up comparison of cardiometabolic risk indicators between patients and controls**

| CMRIs | Patients (n=155) | Controls (n=74) | T-test | | Linear regression  (adjusted for age and sex) | |
| --- | --- | --- | --- | --- | --- | --- |
|  |  |  | **Mean difference (95% CI)** | **P-value**^*^ | **Coefficient estimate** | **P-value**^*^ |
| WHR, mean ± SD | 0.88 ± 0.09 | 0.83 ± 0.08 | 0.05 (0.03 – 0.08) | 0.001 | 0.29 | < 0.001 |
| BMI, mean ± SD, kg/m^2^ | 26.5 ± 5.1 | 24.9 ± 4.3 | 1.6 (0.3 – 2.9) | 0.05 | 0.16 | 0.045 |
| SBP, mean ± SD, mm Hg | 124.9 ± 15.0 | 125.2 ± 13.5 | - 0.4 (- 4.4 – 3.7) | > 0.30 | 0.02 | > 0.30 |
| DBP, mean ± SD, mm Hg | 79.8 ± 8.2 | 77.5 ± 7.6 | 2.3 (0.06 – 4.5) | 0.1 | 0.16 | 0.04 |
| TAG, mean ± SD, mmol/L | 1.3 ± 0.9 | 1.0 ± 0.5 | 0.3 (0.1 – 0.5) | 0.007 | 0.19 | 0.02 |
| TAG/HDL-C ratio, mean ± SD | 1.1 ± 1.0 | 0.8 ± 0.6 | 0.3 (0.1 – 0.6) | 0.01 | 0.19 | 0.02 |
| TChol/HDL-C ratio, mean ± SD | 3.7 ± 1.1 | 3.3 ± 1.0 | 0.4 (0.1 – 0.7) | 0.045 | 0.19 | 0.007 |
| Non-HDL-C, mean ± SD, mmol/L | 3.5 ± 0.9 | 3.2 ± 0.9 | 0.2 (- 0.008 – 0.5) | 0.1 | 0.14 | 0.05 |
| * Corrected for multiple comparisons.  Note  Comparisons are made using multiply imputed data.  Abbreviations: BMI, body mass index; CI, confidence interval; CMRIs, cardiometabolic risk indicators; DBP, diastolic blood pressure; HDL-C, plasma high-density lipoprotein-cholesterol; SBP, systolic blood pressure; SD, standard deviation; TAG, fasting plasma triacylglycerol; TChol, total plasma cholesterol; WHR, waist-to-hip ratio. | | | | | | |
