# Supplementary material for: Time effect on cardiometabolic risk indicators in patients with bipolar disorder: a longitudinal case–control study
Source: Eur Arch Psychiatry Clin Neurosci. 2022 Nov 23;273(5):1191–200. doi: 10.1007/s00406-022-01520-7 (PMC10359211; doi:10.1007/s00406-022-01520-7)
Supplement: Supplementary file 7 — Supplementary file7 (DOCX 23 KB) [file 406_2022_1520_MOESM7_ESM.docx]

**Supplementary table 7. Interaction between the follow-up time and the patient/control-variable with and without adjusting for confounders**

| CMRIs | Participants | Coefficient estimate^*^ | 95% CI | P-value^†^ | Coefficient estimate (adjusted) | 95% CI (adjusted) | P-value^‡^ |
| --- | --- | --- | --- | --- | --- | --- | --- |
| WHR | Controls | - 0.002 | - 0.004 – 0.001 | > 0.30 | - 0.002 | - 0.004 – 0.001 | > 0.30 |
|  | Patients | 0.005 | 0.003 – 0.008 | < 0.001 | 0.005 | 0.003 – 0.008 | < 0.001 |
| BMI | Controls | 0.2 | 0.05 – 0.3 | 0.04 | 0.2 | 0.05 – 0.3 | 0.04 |
|  | Patients | - 0.09 | - 0.2 – 0.03 | > 0.30 | - 0.09 | - 0.2 – 0.03 | > 0.30 |
| SBP | Controls | 0.1 | -0.4 – 0.6 | > 0.30 | 0.1 | -0.4 – 0.6 | > 0.30 |
|  | Patients | 1.1 | 0.5 – 1.7 | 0.003 | 1.1 | 0.5 – 1.7 | 0.002 |
| DBP | Controls | - 0.4 | - 0.8 – - 0.06 | 0.2 | - 0.4 | - 0.8 – - 0.05 | 0.2 |
|  | Patients | 0.8 | 0.4–1.2 | < 0.001 | 0.8 | 0.4 – 1.2 | < 0.001 |
| TAG | Controls | 0.02 | - 0.01 – 0.05 | > 0.30 | 0.02 | - 0.01 – 0.05 | > 0.30 |
|  | Patients | - 0.008 | - 0.04 – 0.02 | > 0.30 | - 0.007 | - 0.04 – 0.02 | > 0.30 |
| TAG/HDL-C ratio | Controls | 0.007 | - 0.02 – 0.04 | > 0.30 | 0.007 | - 0.02 – 0.04 | > 0.30 |
|  | Patients | 0.01 | - 0.02 – 0.05 | > 0.30 | 0.01 | - 0.02 – 0.05 | > 0.30 |
| TChol/HDL-C ratio | Controls | - 0.02 | - 0.06 – 0.01 | > 0.30 | - 0.02 | - 0.06 – 0.01 | > 0.30 |
|  | Patients | 0.03 | - 0.01 – 0.07 | > 0.30 | 0.03 | - 0.01 – 0.07 | > 0.30 |
| Non-HDL-C | Controls | - 0.01 | - 0.04 – 0.02 | > 0.30 | - 0.01 | - 0.04 – 0.02 | > 0.30 |
|  | Patients | - 0.01 | - 0.05 – 0.02 | > 0.30 | - 0.01 | - 0.05 – 0.02 | > 0.30 |
| * Controls´ rows show average annual change in estimates during the follow-up period for the control group. Patients´ rows show estimates for the difference in average annual change between the patient group and the control group.  † P-value adjusted for follow-up time and corrected for multiple comparisons.  ‡ P-value adjusted for age at baseline, sex, and follow-up time and corrected for multiple comparisons.  Note  Comparisons are made using multiply imputed data.  Abbreviations: BMI, body mass index; CI, confidence interval; CMRIs, cardiometabolic risk indicators; DBP, diastolic blood pressure; HDL-C, plasma high-density lipoprotein-cholesterol; SBP, systolic blood pressure; SD, standard deviation; TAG, fasting plasma triacylglycerol; TChol, total plasma cholesterol; WHR, waist-to-hip ratio. | | | | | | | |
